# Usability and utility of a remote monitoring system to support physiotherapy for people with Parkinson's disease

**DOI:** 10.3389/fneur.2023.1251395

**Published:** 2023-10-12

**Authors:** Robin van den Bergh, Luc J. W. Evers, Nienke M. de Vries, Ana L. Silva de Lima, Bastiaan R. Bloem, Giulio Valenti, Marjan J. Meinders

**Affiliations:** ^1^Radboud University Medical Center, Donders Institute for Brain, Cognition and Behaviour, Department of Neurology, Center of Expertise for Parkinson and Movement Disorders, Nijmegen, Netherlands; ^2^Radboud University, Institute for Computing and Information Sciences, Department of Data Science, Nijmegen, Netherlands; ^3^Philips Research, Department of Connected Care and Remote Patient Management, Eindhoven, Netherlands; ^4^Radboud University Medical Center, Radboud Institute for Health Sciences, Scientific Center for Quality of Healthcare, Nijmegen, Netherlands

**Keywords:** Parkinson's disease, physiotherapy, remote monitoring, physical activity, falls, telemedicine, wearable electronic devices, personalized care

## Abstract

**Background:**

Physiotherapy for persons with Parkinson's disease (PwPD) could benefit from objective and continuous tracking of physical activity and falls in daily life.

**Objectives:**

We designed a remote monitoring system for this purpose and describe the experiences of PwPD and physiotherapists who used the system in daily clinical practice.

**Methods:**

Twenty-one PwPD (15 men) wore a sensor necklace to passively record physical activity and falls for 6 weeks. They also used a smartphone app to self-report daily activities, (near-)falls and medication intake. They discussed those data with their PD-specialized physiotherapist (*n* = 9) during three regular treatment sessions. User experiences and aspects to be improved were gathered through interviews with PwPD and physiotherapists, resulting in system updates. The system was evaluated in a second pilot with 25 new PwPD (17 men) and eight physiotherapists.

**Results:**

We applied thematic analysis to the interview data resulting in two main themes: usability and utility. First, the usability of the system was rated positively, with the necklace being easy to use. However, some PwPD with limited digital literacy or cognitive impairments found the app unclear. Second, the perceived utility of the system varied among PwPD. While many PwPD were motivated to increase their activity level, others were not additionally motivated because they perceived their activity level as high. Physiotherapists appreciated the objective recording of physical activity at home and used the monitoring of falls to enlarge awareness of the importance of falls for PwPD. Based on the interview data of all participants, we drafted three user profiles for PwPD regarding the benefits of remote monitoring for physiotherapy: for profile 1, a monitoring system could act as a flagging dashboard to signal the need for renewed treatment; for profile 2, a monitoring system could be a motivational tool to maintain physical activity; for profile 3, a monitoring system could passively track physical activity and falls at home. Finally, for a subgroup of PwPD the burdens of monitoring will outweigh the benefits.

**Conclusions:**

Overall, both PwPD and physiotherapists underline the potential of a remote monitoring system to support physiotherapy by targeting physical activity and (near-)falls. Our findings emphasize the importance of personalization in remote monitoring technology, as illustrated by our user profiles.

## Introduction

Parkinson's disease (PD) is the fastest-growing neurological movement disorder affecting ~6.1 million people worldwide (1, 2). The disease can cause a wide range of motor and non-motor symptoms, such as slowness of movement, tremors, falls, rigidity, cognitive dysfunction, and anxiety. Medical treatment can ameliorate various symptoms, but the complex nature of the disease necessitates multidisciplinary care management (3). One important professional discipline is physiotherapy. Within physiotherapy, persons with PD (PwPDs) learn how to safely maintain activities of daily life, maintain their physical capacity, and train their balance and gait (4, 5).

Important management targets for the physiotherapist are physical activity and fall incidents (4). Physical activity is important to preserve physical capacity and functioning, which are both necessary to continue activities of daily life (6, 7). Performing high-intensity physical activities may even slow down disease progression by stimulating neuroplasticity (8, 9). However, many PwPDs remain or become physically inactive due to problems with gait, balance, and physical functioning (10, 11). Fall incidents are also important because they can negatively impact a person's quality of life (12), for example, by instilling a fear of renewed falls or by causing a (hip) fracture (13–15). A vicious cycle between physical activity and fall incidents can occur when a fear of falling leads to reduced physical activity (16), and reduced physical activity leads to increased fall risk because of general weakness (12). Conversely, promoting physical activity through a therapeutic exercise regime may paradoxically increase falls, which, by definition, occur more often in those who are physically more active.

Accurate assessment of physical activity and falls during common daily activities would be a tremendous help for the physiotherapist to create individually tailored treatment plans. For example, a fall caused by festination requires a different treatment plan than a fall caused by muscle weakness. Usually, physical activity and falls are assessed with short questionnaires, in-clinic motor tasks, or self-reports (4, 12). However, in-clinic physical assessments often give a false impression as PwPDs typically behave differently in the clinic than in their own homes (17, 18). Self-reports or questionnaires can also be burdensome and are subject to recall bias, even more so among those with coexistent memory or other cognitive problems (19, 20).

By contrast, wearable sensor data can provide accurate, continuous, and objective information to support physiotherapy. Wearable sensors are often present in accelerometers and gyroscopes which are unobtrusively packed in, e.g., smartwatches and smartphones (21). Their size and shape make them a feasible option to be worn in daily life (22). Even for prolonged periods, ranging from 6 weeks up to 2 years, excellent compliance can be achieved by monitoring PD using a smartwatch or sensor (23–25). Additionally, wearable sensors can be used to quantify both physical activity and falls in daily life (26–28). Despite their feasibility and accuracy, only a few studies have tested the application of wearable sensors in physiotherapy practice. Preliminary findings show that it is feasible to capture sensor data during in-clinic training sessions and that the data can support balance training through sensor-based biofeedback (29, 30). Furthermore, physical activity training could be remotely supervised by streaming vital sign data to a tele-coach (9, 31). However, to advance implementation in clinical practice, more studies are needed in which both physical activity and fall data are combined into a single system that is rigorously tested in everyday life.

In this study, we designed a remote monitoring system for physical activity and falls. The system consisted of a necklace tracking movement, an app for PwPDs to review recorded activities and manually add undetected ones, and a physiotherapist app to review any incoming data. We evaluated the usability and utility of the system to support physiotherapy for PwPDs. We employed an iterative design process in which we closely collaborated with both physiotherapists and PwPDs and tested the system twice in practice for 6 weeks.

## Materials and methods

### Study design and participants

In an iterative process, we developed and evaluated a remote monitoring system consisting of a wearable sensor and mobile app, further described under the “Materials” section. The study consisted of two pilots which were 1 year apart (2017 and 2018) and which spanned 6 weeks each. In both pilots, PwPDs used the remote monitoring system and discussed the collected data during three regular treatment sessions with their physiotherapists. Before pilot 2, the system was updated according to user feedback from pilot 1.

Pilot 1 included nine physiotherapists and pilot 2 included eight physiotherapists, one of whom also participated in pilot 1. We recruited the physiotherapists via ParkinsonNEXT, an online platform that facilitates research participation for healthcare professionals and PwPDs in the Netherlands. Physiotherapists were eligible if they were members of ParkinsonNet, a network of healthcare professionals specialized in PD (32).

Subsequently, the included physiotherapists recruited PwPDs from their own practice. The inclusion criteria for PwPDs in pilots 1 and 2 were largely similar. For both pilots, participants needed to be diagnosed with PD by a neurologist or movement disorder specialist, be at least 30 years of age, and receive physiotherapy for PD for at least four weekly sessions within 6 weeks after study enrollment. In pilot 1, we aimed to include 20 PwPDs who were required to own and (cognitively) be able to use a smartphone with an Android operating system ≥ 5.0. In pilot 2, we aimed to include 25 PwPDs of whom 20 were required to own or use a smartphone and five were not. These five PwPDs could test the wearable sensor without the smartphone app. Among these 25 PwPDs, we aimed to include at least 10 PwPDs who had fall or balance problems, as judged by the physiotherapist. The study was conducted in compliance with the Ethical Principles for Medical Research Involving Human Subjects, as defined in the Declaration of Helsinki, and was approved by the local ethics committee (CMO regio Arnhem-Nijmegen; file 2017-3382). All participants gave written informed consent before enrollment.

We adhered to the Consolidated Criteria for Reporting Qualitative Research checklist for reporting the qualitative part of our study.

### Materials

The remote monitoring system, i.e., the Vital@Home system, consisted of a wearable sensor in the form of a necklace (the “GoSafe”), a Wi-Fi hub, a custom-developed Android smartphone app for PwPDs, and a custom-developed Android tablet app for physiotherapists. We created the prototype of this system based on recommendations for physiotherapy in PD (4), prior experiences with wearables and physiotherapy within the research team, and technical feasibility. For technical feasibility, four PwPDs used this prototype at home for 2 weeks to pilot test the interaction with the patient app. Consequently, we made minor adjustments to the user interface to improve its usability. Then, the system was evaluated in the two pilots reported here. In the next section, we have described the system as it was used in pilot 1. Table 1 describes the changes made to the system after pilot 1 and the desired changes to the system mentioned in pilot 2.

**Table 1:** The features of the Vital@Home system across both pilots as well as desired future features.

	**Pilot 1**	**Added in pilot 2**	**Future wishes**
Physical activity	• Walking detected • Self-report others • Progress toward physical activity goals displayed	• Feedback on wearing compliance • Number of steps displayed	• Detect more diverse activities (biking, household, swimming) and fewer self-report • Detect activities shorter than 10 min • Assign intensity level to all activities • Personalized activity goals • Real-time data transmission to the app
Falls	• Daily questionnaire at 18:00 • Manual report during the day through the app	• Falls detected by the necklace • Daily questionnaire only when fall detected • Feedback on step time and step time regularity to assess fall risk • Freezing of gait diary	• Automatic alarm when the wearer does not respond • Balance measurement • Elaborate fall risk assessment based on algorithms • Automatic FOG detection • Daily life gait and transfer analysis extended (e.g., stride length and walking speed)
Medication	• Daily manual medication registration	• Option to enter daily medication scheme and set reminders • Option to report individual medication intakes by responding to medication reminders	• Personally adjustable medication dose • All manual registrations can be corrected
Additional features		• Personal exercise program	• More in-person guidance and support on operating the system • Option to comment on data, e.g., *moved less because of bad weather*
Technical components	• Necklace • Wi-Fi hub • V@H patient app • V@H physio app	• Necklace • Wi-Fi hub • V@H patient app (optional) • V@H physio app	• Necklace or smartwatch (choice) • No Wi-Fi hub • V@H app (optional)

#### The GoSafe necklace

**Figure 1:**
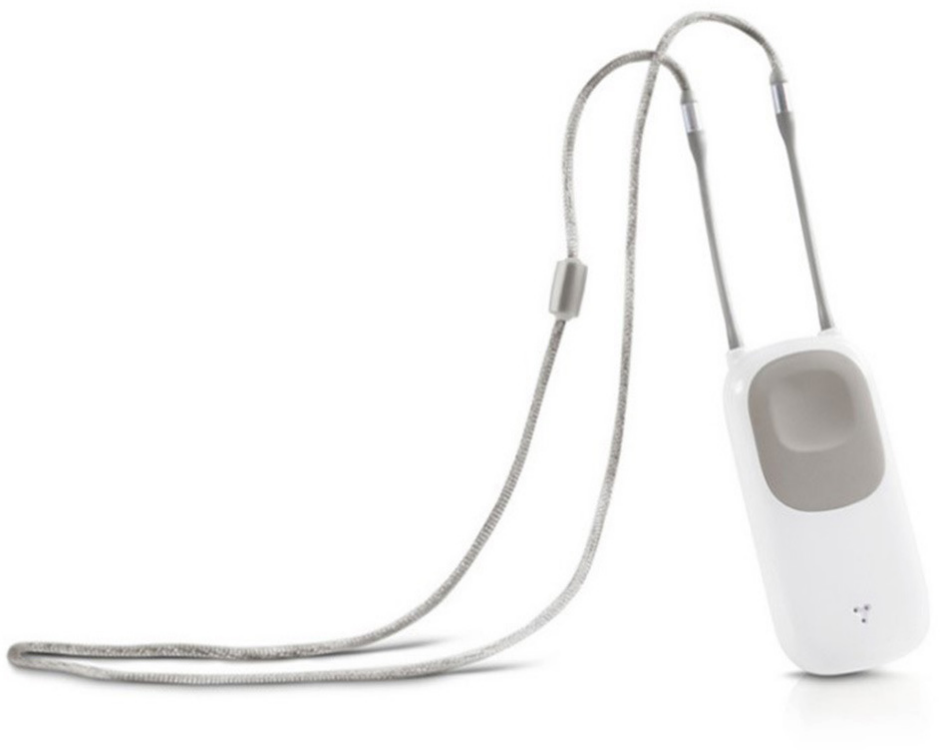
The Philips lifeline GoSafe necklace.

The GoSafe necklace (Figure 1; Philips Lifeline, Framingham, MA, USA) is a wearable sensor that is commercially available in the United States as part of a medical alert service. The necklace contains multiple sensor types, including an accelerometer, a barometer, and a GPS sensor. We derived the person's physical activity and fall incidents from the sensor data using proprietary algorithms developed by Philips Research (33, 34). The algorithm is based on continuously collected accelerometer data and walking bouts of at least 10 min. Fall incidents were detected based on continuously collected accelerometer and barometer data. Data collected with the GoSafe were streamed via the Wi-Fi hub to a secured Amazon server located in Germany, managed by Philips. The GoSafe necklace has received FDA approval. A European Declaration of Conformity was provided for use in this study.

#### Vital@Home patient and physiotherapist apps

The Vital@Home apps were developed as part of a European Institute of Innovation & Technology (EIT)-funded collaboration between TU Berlin, Curamatik, Radboudumc, Philips Research, and University College London. The display language of both apps was Dutch for the current study, although an English version was also available.

**Figure 2:**
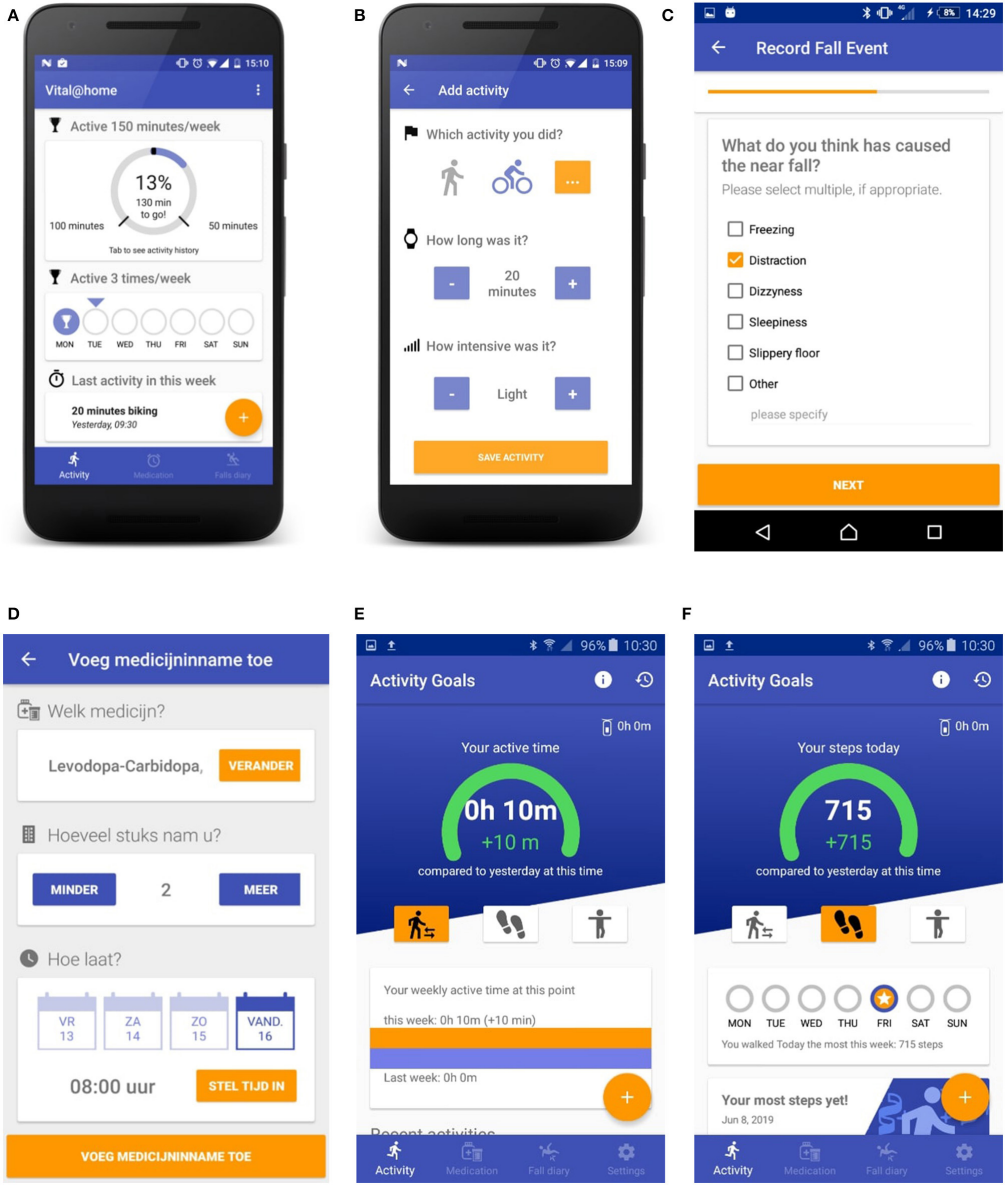
The Vital@Home application for persons with PD in pilot 1 **(A-D)** and pilot 2 **(E, F)**, including the homepage of the app displaying progress toward physical activity goals **(A)**, the manual entry of activities **(B)**, a part of the fall questionnaire **(C)**, the medication registration **(D)**, and the reworked activity **(E)** and step count **(F)** homepage for pilot 2. *Translation of 2d: Add medications (top); Which medication? How many did you take? At what time? (questions in the middle); Confirm medication intake (bottom).

The app for PwPDs ran on an Android smartphone and contained three sections: physical activities, falls, and medication intake (Figure 2). For physical activities, the app provided an overview of all gait bouts detected by the GoSafe necklace. In addition, users were encouraged to manually enter sports activities that were not automatically detected, such as cycling or swimming. For all manually entered activities, users were asked to report the type, duration, and level of exertion using the BORG Rating of Perceived Exertion scale (35). The app gave feedback on how close users were to reaching their daily and weekly activity goals. These activity goals were determined by the PwPD and physiotherapist together based on clinical judgment and personal preferences. The app automatically prompted the participant with a questionnaire at the end of the day (18:00 h) asking for verification of any detected falls and followed up with questions about the context of the fall incident. These questions were based on the falls diary included in the European Physiotherapy Guideline for Parkinson's Disease (4) and included questions about the self-perceived cause of the fall incident, environment, and motor state (off/on/on with dyskinesias). In addition, users could manually start this questionnaire at any time of the day to register near-falls or falls. Users could also manually register their medication intake during the day. All the gathered information was accessible to the PwPDs in the app.

The app for physiotherapists ran on an Android tablet and could display the information from their client during a treatment session (Supplementary Figures 1–7). The physiotherapist app contained an overview of all recorded physical activities and the progression toward the weekly goals. It also showed the number of (near-)falls and the answers to the fall-context questionnaire. The app displayed patterns over time, but could also show individual registrations of physical activities and falls. The physiotherapist could only access the sensor data during the treatment session by using the physiotherapist app to scan a QR code displayed on the app of the PwPDs. For pilot 2, some participants did not use the app and their physiotherapist could always see the data.

### Procedures

The procedures for each pilot were largely similar. In both pilots, physiotherapists were recruited and trained on study procedures, study assessments, and usage of the Vital@Home system. Then, each physiotherapist recruited two or three PwPDs within their own practice. These PwPDs were scheduled to have at least four weekly physiotherapy sessions after study enrollment. Participants were prospectively followed for at least 4 weeks with a maximum of 6 weeks. During the first study visit, physiotherapists conducted a clinical assessment (see “Outcomes and analyses” section) and instructed PwPDs on the usage of the Vital@Home system. After the first study visit, the PwPD wore the necklace at home during the day and charged it during the night. Preferably, a minimum of 8 h of sensor data were collected per day to provide enough information. The PwPD and physiotherapist discussed the collected information during three consecutive treatment visits. A member of the research team was available for technical support throughout the study duration.

After the fourth visit, a researcher interviewed each physiotherapist face-to-face and each PwPD via telephone for 20–40 min to capture their experiences using the Vital@Home system. LE (man) and AS (woman; both PhD students) conducted all interviews after receiving qualitative interviewing training. There was no relationship between the interviewer and the participants before the interview, except for any contact necessary for enrolment and participation in the study. The interviews were semi-structured, meaning that the interviewer used a guide to conduct the interview but was free to diverge from the guide and go more in-depth when the interviewee expressed an interesting or elaborate opinion on a topic. The guide covered five topics: general experiences of using the system including future wishes, usability of specific features, utility of specific features, technical functioning, and reliability of the registrations. The interviews were audio recorded and transcribed verbatim. PwPDs also completed an online version of the System Usability Scale (36).

**Figure 3:**
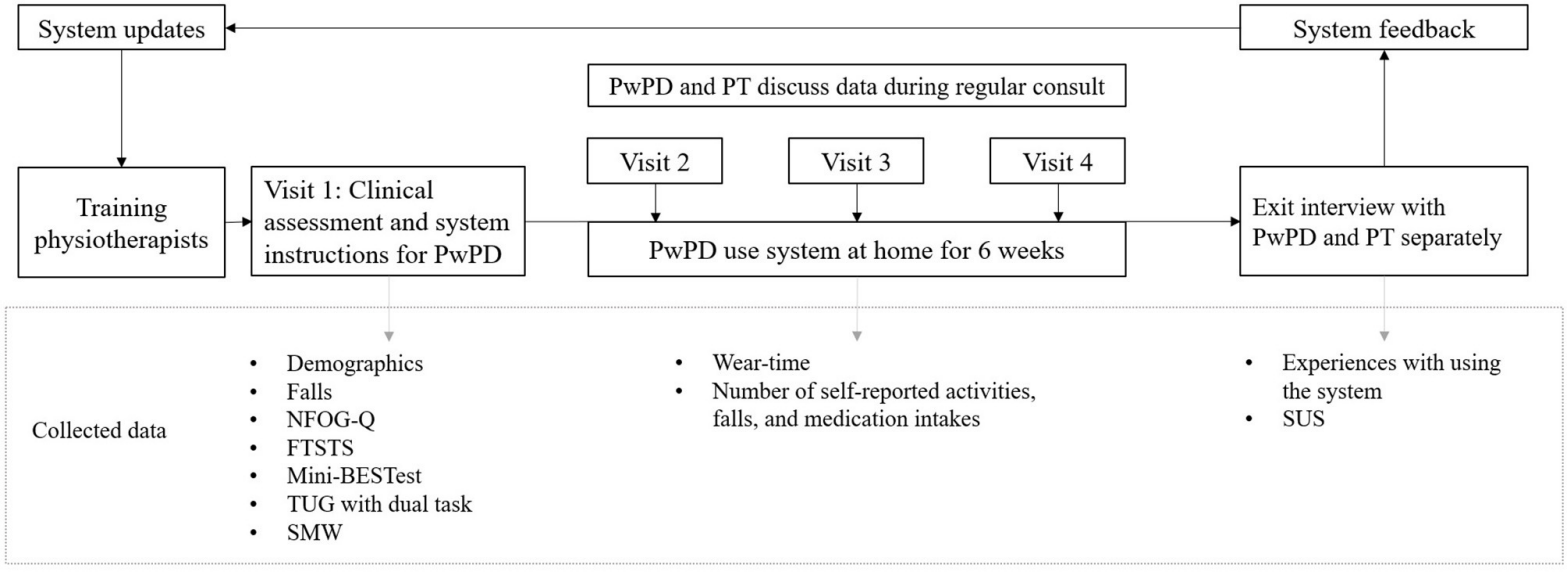
Overview of study procedures and measured outcomes. The procedures were completed twice. PT, physiotherapist. PwPD, persons with Parkinson's disease. NFOG-Q, New Freezing of Gait Questionnaire, self-reported amount of FOG moments in the past month. FTSTS, Five Times Sit To Stand, measures balance during transfers. Mini-BESTest, Mini Balance Evaluation Systems Test, measures static and dynamic balance. TUG, Timed Up & Go, measures functional mobility. SMW, Six Meter Walk, measures comfortable walking speed, for pragmatic reasons shortened version of 10 Meter Walk. SUS, System Usability Scale, measures perceived usability of the system.

Based on the results of pilot 1, improvements and new features were implemented in the Vital@Home apps (Table 1). The updated version of the app was tested in pilot 2 with another group of physiotherapists and PwPDs. One physiotherapist and two PwPDs participated in both pilots. All participants in pilot 2 adhered to the same procedure as in pilot 1 to test the system in practice. The only three differences were: the updated system version, PwPDs wearing the necklace also at night, and the GoSafe only option for participants without a smartphone. In pilot 2, participants charged the necklace whenever needed instead of specifically during the night. Figure 3 gives an overview of the study procedures and collected data.

### Outcomes and analyses

In both pilots, we collected demographic and clinical assessment data of PwPDs to characterize our sample. The assessments were performed by physiotherapists during the first study visit and included a history of falls, the Mini-BESTest including the Timed Up and Go test with and without dual task (37), the presence of freezing according to the New Freezing Of Gait Questionnaire (38), the Five Times Sit To Stand test to assess balance and fall risk (39), and the Six Meter Walk test to measure comfortable walking speed, which, for pragmatic reasons, is a shortened version of the 10 Meter Walk test (4).

As the primary outcome measure, we report the qualitative experiences of PwPDs and physiotherapists who used the system. We applied thematic analysis to the anonymized transcripts of the interviews with PwPDs and physiotherapists (40). First, two researchers read all transcripts and independently coded meaningful sections of the first 20 interviews. Any discrepancies between the coded segments were discussed and resolved. Subsequently, each researcher independently coded half of the remaining interviews which were checked by the other. We coded deductively based on five themes derived from the interview guide: usability, utility, technical functioning, reliability of the registrations, and suggestions for improvement. However, we also allowed for new themes to be inductively identified in the data. We generated non-overlapping themes and subthemes based on our deductive and inductive coding process aiming for internally consistent themes that each captured a unique aspect of the dataset. We constantly compared new codes and themes against codes and themes we already had and periodically went back to our already created codes and themes. We discussed the phrasing and content of themes as well as the thematic structure within the research group to ensure the high quality of the work. We kept track of the analytical process and researcher decisions with memos. The research team agreed upon the final version of the thematic structure. ATLAS.ti version 8 was used for the qualitative analysis (41).

As secondary outcome measures, we collected data on compliance in two forms: the number of days with at least 8 h of sensor data collected across the minimal study duration of 28 days and the number of self-reports entered in the app. We also computed the score on the System Usability Scale (SUS, range: 0–100) (36). We report descriptive statistics of sample characteristics, compliance, and SUS as calculated with R Statistical Software v4.1.3 (42, 43).

Finally, we drafted user profiles based on the interviews to understand when, why, and for whom the monitoring system can add value. User profiles represent typical user characteristics such as skills, motivations, behaviors, needs, and goals of the users (44). They capture common patterns or similarities in these characteristics to create a better understanding of system users. During the interviews, physiotherapists were asked to which patient population they thought the system would add value. We corroborated their answers with the interview data from PwPDs, which contained information on the user profile domains. The first author drafted a first outline of the user profiles by grouping participants based on the interview data regarding digital literacy, behaviors, needs of the person, and the perceived utility of the system. Thereby, the user profiles were grounded in recurrent statements across interviews with participants. The profiles were then discussed with other members of the research team (LE, NdV, MM, and RvdM) until a consensus was reached.

## Results

We included nine physiotherapists and 21 PwPDs in pilot 1 and eight physiotherapists and 25 PwPDs in pilot 2. Eleven out of the 25 PwPDs in pilot 2 used the GoSafe only, either because they did not possess a smartphone (*n* = 6) or their smartphone version was not compatible with the app (*n* = 5). In pilot 1, three PwPDs dropped out during the study because the system was too complicated for them. They were included in the interview data. No PwPD dropped out during pilot 2. Table 2 shows the demographic and clinical characteristics of all PwPDs.

**Table 2:** Demographic and clinical characteristics of the persons with Parkinson's disease participating in the two consecutive pilot studies.

**Variable**	**Unit of measurement**	**Pilot 1 *N* = 21**	**Missings**	**Pilot 2 *N* = 25**	**Missings**
Gender	No. of men	15 (71%)	0	17 (68%)	0
Age	Years	65.5 ± 8.0	0	68.7 ± 9.4	0
Hoehn and Yahr stage	≤ 2	5 (50%)	11	15 (83%)	7
	3	5 (50%)		2 (11%)	
	≥4	0 (0%)		1 (6%)	
Time since diagnosis	Years	3.5 (1-17)	11	^*^	25
Medication usage	Levodopa	20 (95%)	0	23 (92%)	0
	Dopamine agonist	8 (38%)		2 (8%)	
	Other	5 (24%)		2 (8%)	
Experienced ≥1 near-fall(s) in past 12 months	Yes	1 (5%)	2	13 (59%)	3
Experienced ≥1 fall(s) in past 12 months	Yes	4 (20%)	1	16 (64%)	0
Experienced freezing of gait (NFOG-Q)	Yes	6 (29%)	0	6 (24%)	0
FTSTS	Time (seconds)	12.5 ± 4.6	0	13.7 ± 4.9	1
Mini-BESTest	Average score	24.1 ± 3.6	3	22.8 ± 4.2	3
	Score ≤ 22	5 (28%)		9 (41%)	
TUG with dual-task	Time (seconds)	12.5 ± 5.7	0	13.2 ± 12.2	0
SMW	Walking speed m/s	1.30 ± 0.33	0	1.21 ± 0.24	0

Data are presented as mean ± SD or *n* (%), except for time since diagnosis (median and range). Calculations are based on valid data. NFOG-Q: New Freezing of Gait Questionnaire, self-reported amount of FOG moments in the past month. FTSTS: Five Times Sit To Stand, measures balance during transfers. Mini-BESTest: Mini Balance Evaluation Systems Test, measures static and dynamic balance; scores ≤22 indicate significant balance problems. TUG, Timed Up & Go, measures functional mobility. SMW, Six Meter Walk, measures comfortable walking speed. SD, Standard Deviation.

^*^Not assessed during pilot 2.

**Figure 4:**
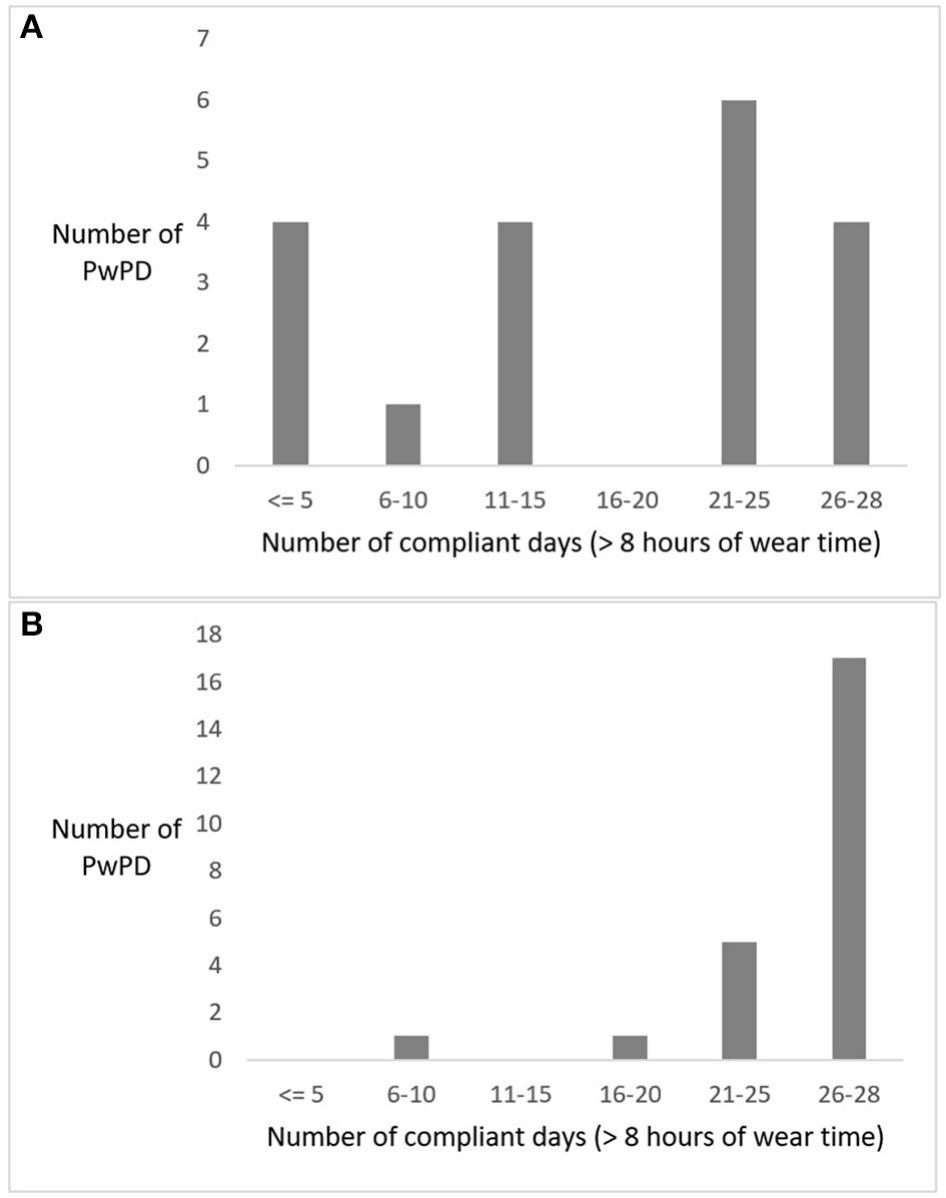
Frequency distribution of the number of compliant days for all persons with Parkinson's disease (PwPDs) wearing the GoSafe necklace in pilot 1 **(A)** and pilot 2 **(B)**.

Compliance with wearing the sensor varied considerably in pilot 1, with 9 participants having 15 or fewer compliant days out of 28, while 10 participants had more than 21 compliant days (2 missing, Figure 4). In pilot 2, compliance was higher with 22 out of 25 participants having 21 or more compliant days (1 missing, Figure 4). In pilot 1, PwPDs created 1,893 medication reports and reported 30 (near-)falls in 6 weeks (at the time of writing, these data were unavailable for pilot 2). The SUS score among PwPDs was higher in pilot 1 (*M* = 63, *SD* = 16) compared to pilot 2 (*M* = 54, *SD* = 25).

### User experiences with the system

Initially, we started the qualitative analysis with five themes. However, throughout the analytical process, we identified two themes that best characterize the users' experiences with the system: the usability of the system and the utility of monitoring information. Statements regarding the technical functioning and reliability of the registrations gave context to the usability and utility but were not clearly demarcated themes on their own. The future wishes are separately listed within the overview of system features (Table 1). Some are also highlighted under subthemes when applicable. Quotes illustrating the subthemes are given in-text and in Table 3. The results of pilots 1 and 2 are jointly discussed as feedback was highly comparable.

**Table 3:** Quotes reflecting the user experiences with the Vital@Home remote monitoring system.

Usability	Operating the system in daily life	Pilot 2 PwPD 3: But apart from that, the necklace, so to speak, around the neck did not bother me at all. I just kept it on day and night and it didn't bother me at all
		Pilot 1 PwPD 10: Yes, the size of the device and the cord were not pleasant
		Pilot 1 PwPD 11 about Wi-Fi hub: That was annoying at times, because I forgot about it. And when you're at home it's all fine, but when you leave it's a bit more complicated. Then you have to think about it
		Pilot 2 PwPD 1 about the app: Yes, that was clear. That is not a problem
	Digital literacy and support	Pilot 1 PT 2: In the beginning, I found it quite a hassle, especially for the patient. You have to explain, they don't quite understand, and I don't quite know myself either. So, it took a while… but after two weeks you get used to it
		Pilot 1 PwPD 4: Yes, that, perhaps, it [digital support] is not so easy from a distance […] That perhaps you should discuss together in a kind of circle conversation, what the questions exactly mean and what you can do. It is so distant
		Pilot 2 PT 3: I think I would go for that [GoSafe only] more because then patients just have to carry it and not add any additional actions and then when they come to me, we can look at their app together and then retrace or analyze or discuss things, rather than them having to do all that themselves
		Pilot 1 partner of PwPD 3 managing the app for him: So we did sit on the sofa together in the evening and then we entered everything, because I wanted my husband to know what I was doing. And then I would say: shall I [enter] so many minutes step or so many minutes… so that it all comes from him, so to speak
	Technical prerequisites	Pilot 1 PwPD 12: Yes, that [walking detection] is pretty good, because when we went for a walk, my wife came along every time, we would check beforehand, I'll call it we're leaving five to nine-thirty, and I'll be home at a quarter past ten, that's how long we've walked. And, there might be a minute or two or three in it altogether, but otherwise, he's goo
		Pilot 1 PwPD 4: And… yes, the annoying thing is that then you type in the data and you see that you have made a mistake, but you cannot correct it
Utility	Physical activity monitoring	Pilot 1 PwPD 13: For example, when I'm doing my household, I go upstairs, I go downstairs again, it doesn't register that. And if I for example vacuum my whole house, yes, I find that quite an effort, because then I have to rest now and then. But it doesn't register that at all
		Pilot 1 PT 2: So, I notice that it works in this way for the patients to be more active, to realize more that exercise is important. And yes, also for themselves, because I didn't encourage them to move more, I didn't say anything about that, because I always say you're doing well, but they just started setting some kind of goals. Like oh, but then [I] want to… because they know that they can see it [sensor data] back with me. So, it does work that way
		Pilot 1 PwPD 1: And then with three days I had closed the circle and I could finish the week with 200 min extra, so to speak. And that gave me a good feeling. So, I was constantly challenging myself
	Falls monitoring	Pilot 1 PT 4: And you can see, also with the falling, when it goes wrong and if that has to do with the medication or with other activities. Whether they have become very active and then fall […] So, I really do see potential in that
		Pilot 1 PwPD 9: Was that last question… Have you fallen today? I have not fallen during that whole period, I have never fallen
	Role of the system in consult	Pilot 1 PT 2: I actually already knew […] how much someone moves and how often they exercise. You want to have insight into that. And yes, that was actually just a confirmation. But that's not to say that it doesn't work, it just hasn't added anything to my treatment
		Pilot 2 PT 2: I don't know if that could be that you, speed indeed, but also a certain rhythm, or that people change speed, so whether people start festering or people start freezing, if indeed you could see that

PT, physiotherapist; PwPD, person with Parkinson's disease.

### Usability of the system

The usability of the system, i.e., its ease of use, was overall rated positively. We identified three subthemes that characterize this theme. First, participants described how they *operated the system in daily life*. Most PwPDs mentioned that wearing the necklace was not burdensome. Some found the cord annoying, especially during the night, but most PwPDs were positive about its ease of use. While most PwPDs were not bothered by the necklace being visible to others, some left the necklace at home when they left the house so as to not raise any questions. In the future, the necklace's battery life of this prototype should be increased and fluctuate less, as these fluctuations made participants uncertain about how long the battery would last that day. A clear indicator of the remaining battery life could take away much of this uncertainty.

*Pilot 1 PwPD 1: You get up in the morning and after showering you put it around your neck and forget about it*.

*Pilot 1 PwPD 2: Look, but if you go among people then, well, I leave it [the necklace] at home pretty quick. Then I say it has worked enough for today […] you also don't want to make yourself look more disabled than you already are*.

*Pilot 1 PwPD 3: So if it was charged then it was a constant green light, but then you don't know if it's really already properly charged and with a smartphone you can just see how full it is*.

The Wi-Fi hub, necessary for data transfer, puts little strain on the PwPDs and their caregivers as it was often permanently placed in the charger and required little further attention. Participants were instructed to carry the hub with them when leaving the house for 3+ h, which was no problem for most of them.

The user interface of the app was regarded as very clear, intuitive, and user-friendly by both PwPDs and physiotherapists. Only a few PwPDs had issues with understanding the different screens.

*Pilot 1 physiotherapist (PT) 1: That's a clear screen. Yes, clear. At a glance, you could see that*.

However, many PwPDs from pilot 1 mentioned that registering their medication intake in the app was not user-friendly. For example, medications had to be entered manually each day and mistakes were not correctable. In pilot 2, the medication function was thoroughly revised so that a medication schedule was repeated throughout the weeks, which could be confirmed with a single button, only requiring deviant medication intakes to be manually entered. In addition, automatic reminders of medications were sent. As many PwPDs have stable medication schemes, this was experienced as very helpful.

*Pilot 2 PwPD 1: But, the drugs, on the other hand, that was great. (What was good about that?) Well, pre-programming, of course, with time. It's just confirming and that's it. Last year, I think you had to fill everything in again*.

The second subtheme regarding the usability of the system was the importance of the *digital literacy* of the participants and the *support* offered by the environment. In pilot 1, all participants had to manage the necklace, hub, and app, which was no problem for technically adept participants. However, some PwPDs and physiotherapists struggled with the technology. For example, they did not understand when the devices were connected and how they could see them. The technical support offered throughout the study was appreciated and used by participants. The assistance of the partner also helped to retain less digitally skilled PwPDs in the study.

*Pilot 1 PwPD 4: I was stuck with the fact that those things made a lot of mistakes in the beginning; it was all uncomfortable. And I didn't understand yet how it all fits together logically. That just takes a few days to get used to*.

*Pilot 1 PwPD 5: It is more difficult for older people. They already have problems with a computer, so sometimes you don't understand it, or something. But yes, you can call you, you can call the physiotherapist. So you do have enough backing if you want to know something*.

Despite the offered support, the system proved too difficult for some PwPDs due to suspected cognitive impairments and insufficient experience with digital technology. For example, an older caregiver mentioned that monitoring the connection of the Wi-Fi hub as well as the battery of the necklace and smartphone was too much to manage at the same time.

*Pilot 2 partner of PwPD 2: I once looked in the beginning [in the app], but you know? Our age is pretty high. We're 79 and 80, so we didn't grow up with all that stuff […] also with keeping an eye on the fact that it has to be charged. Then, there are three different things - your phone and the device and the Wi-Fi - that you have to keep an eye on [Partner] can't do that anyway, but anyway, you're often busy with all sorts of things and then you forget about it*.

Finally, participants mentioned *technical prerequisites* as being important for the usability of the system, such as data being accurate, automatically recorded, and correctable. The participants stated that the system accurately detected walking activities. However, the system required other activities such as housekeeping and cycling on a home trainer to be manually entered. The possibility to manually register non-detected activities was valued by some participants but was typically experienced as burdensome as participants continuously had to remember the duration and intensity of their activities. Furthermore, PwPDs could make mistakes when manually entering activities and medication intakes. For example, sometimes the data transfer from the sensor to the app spanned more than a day, making PwPDs believe that the activity had not been recorded. They would manually enter the activity which resulted in double registration of activities once the sensor data became visible. PwPDs could not correct these mistakes that caused some frustration. In the future, PwPDs desired the automatic detection of more diverse activities and real-time data transfer.

*Pilot 1 PwPD 6: Initially, in the first week, I entered my own walks, because it didn't indicate that. But after a week, then all of a sudden it was all in there, with the result that it was all in there twice of course*

*Pilot 1 PwPD 3 and partner: We still do as much or as little […] because, then, that app says if I fill it in wrong then that round was closed again and then it said: completed. And then I think: yes, that is nonsense actually because that is not correct at all*.

### Utility of monitoring information

The utility, i.e., added value, of the monitoring information can be described by three subthemes. First, the monitoring of *physical activity* elicited mixed reactions by PwPDs and physiotherapists. Some PwPDs stated that tracking physical activity was not adding value to them because they were already aware of how active they were. Also, several PwPDs and physiotherapists stated that the data lacked detail to draw strong conclusions. For example, some PwPDs mentioned that walking up and down the stairs was quite challenging for them. They wondered why such short bouts of activities were not displayed in the app.

*Pilot 1 PwPD 7: No, because, in that situation [daily life], I think I know what I'm moving and what I'm doing, I still work fulltime, so I know exactly what I'm doing and what not*.

*Pilot 2 PT 1: And, certainly in this target group, I think, because I think that, for some people, for example, walking for eight minutes can be quite a lot, and if that doesn't actually count, then that's a shame. Then it actually works against them, so to speak*.

In contrast, numerous PwPDs stated that the system motivated them to move more. Seeing their data made them aware of their activity levels and motivated them to reach their weekly goals by becoming more active. Some participants even became so enthusiastic about tracking their physical activity that they, after the study had ended, bought commercially available smartwatches to continue self-monitoring. For some physiotherapists, the objective data formed a pleasant confirmation of the assumed physical activity level of the PwPDs at home. In pilot 2, a video-based exercise section was added to the patient app (Table 1) so that PwPDs could have video examples of how to exercise at home. The exercises were purely informative and not specifically monitored as our study was not concerned with the remote delivery of physiotherapy sessions. The exercise examples in the app were appreciated by some PwPDs, and a couple of physiotherapists found it useful to see which at-home exercises were being completed. However, this feature held limited utility as many PwPDs already knew how to complete the exercises or were using a different app provided by the physiotherapist.

*Pilot 1 PwPD 8: Yes, it certainly works; it certainly works for me. Yes, really, because then you are forced to face the facts, you think: yes, I must exercise more. Because you sometimes postpone it because you often have difficulty with it, because walking is sometimes more difficult for me. Also, because your balance is not so good anymore, and then you think: yes, it is best for me actually, that I do it, to move*.

*Pilot 2 PwPD 3: Well, I bought myself a wristband now […]. Because if I haven't moved enough, it means I have to walk around the block in the evening, because I plan to take so many steps a day*.

*Pilot 1 PT 2: It does add that you get confirmation if someone is indeed exercising, if someone is moving or not*.

Second, the monitoring of *falls* was mentioned as being important by both physiotherapists and PwPDs. One advantage was that PwPDs were made more aware of the importance of (near-)falls. In addition, physiotherapists liked the insight into the context and timing of a fall, e.g., knowing how physically active people were or linking the fall to medication intake. However, the fall-related section of the system was not relevant for many PwPDs, as they did not experience any (near-)falls during the 6 weeks of use of the system.

*Pilot 1 PT 3: But, with that fall agenda, I found that, just to make people already aware of those near-fall incidents… because you do mention that, but… much more often consciously, like, “oh, if I fall backwards or if I want to grab a.. and find support against the wall.” So, I thought it made sense anyway to make patients more aware*.

*Pilot 2 PT 2: Then, it would be nice to have a combination of: gosh, what did they do that day? Look, if someone feels like they haven't been doing all that much, but we think, hey, they're overexerting themselves and that's why they're falling; yeah, I think you can get some nice feedback on that. And you just have, when people wear it for a longer time and people actually fall more often; yes, then you just get an overview of hey, then and there and then and there*.

Third, both physiotherapists and PwPDs mentioned the *role of the system in the consultation*. As a benefit, physiotherapists stated that the objective sensor-based information and the subjective self-reports provided them a view and insight into the at-home activities and daily life functioning of the PwPDs. Discussing the information provided them with more structure during the consultation to systematically address the topic of physical activity and falls. However, the added value of the system was limited for several physiotherapists and PwPDs because the therapy goals were already clear and manageable, meaning there was limited room for improvement of therapy based on the additional information.

*Pilot 1 PT 3: But, usually you just ask about it [physical activity], but to really have it come back so systematically, and that it is also even more important what they do at home, to make them even more aware of it, I thought it was very nice to do it this way*.

*Pilot 1 PwPD 9: We didn't go all that deep into it, but then again, if there were no problems then you don't have anything to talk about, do you?*

Importantly, many PwPDs highly value the relationship and interaction with the physiotherapist. Many PwPDs, therefore, enjoyed discussing the data with their physiotherapist. Several PwPDs felt extra motivated to move more to show the physiotherapist how active they had become.

*Pilot 1 PwPD 8: Yes, that [discussing the data] is always positive, of course. But that happens anyway, because we had a conversation about it every time. Because it also stimulates to undertake more activities, doesn't it?*

*Pilot 2 PT 1: And every week I took the tablet and looked at it. They liked that, because they are participating, so then it's kind of… Yes, they liked that*.

The physiotherapists noted that the system could become more relevant within the consultation (Table 1). For example, they desired more advanced analyses of gait and balance parameters to adjust therapy. In pilot 2, we added a gait pattern analysis section to the app. This section provided physiotherapists with a −3 to +3 score reflecting the quality of gait of the PwPDs. The interpretation of this score was yet unclear to physiotherapists, but the potential use of such analyses was apparent to them.

*Pilot 2 PT 3: Yes, because the step length, step frequency are things that I would like to get though, if there is a change in that*.

### User profiles

We drafted three user profiles that describe how a remote monitoring system can add value to physiotherapy (Table 4).

**Table 4:** User profiles of persons with PD receiving physiotherapy drafted from interviews.

		**Profile 1**	**Profile 2**	**Profile 3**	
	Stage of PD	Early-Mid	Mid	Mid-Late	
	Digital literacy	+++	++	+	
	Cognition	+++	+++	+	
	Physical activity level	+++	++/+	+	
	Fall incidents	Absent	Rarely, or near-fall experiences	More frequent	
	Physiotherapy goals	• Early identification and treatment of issues, e.g., inactivity or fear to move • Potential to slow disease progression	• Desires to move more • Challenge to keep motivation high • Treat issues with balance to prevent falls	• Treat issues with balance to prevent falls • Keep functional mobility to perform day-to-day tasks	
**Persons without physiotherapy-related problems**	Utility of the system	• Keep motivated to stay physically active • Prevent major issues by proactively screening for beginning problems (flags), e.g., through an in-depth analysis of gait parameters • Track disease progression to know when to initiate treatment, thereby preventing overtreatment	• Increase and maintain higher levels of physical activity • Discuss data with the physiotherapist to raise awareness of the importance of physical activity and falls, and to support understanding of own PD • Track disease progression to set treatment goals, and easily share information among healthcare professionals	• Collect objective and accurate data about mobility and balance at home for physiotherapist • Context questionnaires can provide insight into falling circumstances	**Persons for which monitoring is too burdensome or technically too complicated**
	Usability of the system	• Operates sensor and app to (self-) monitor at home independently • Analyses data alone and together with physiotherapist	• Operates sensor and app to monitor at home with support • Interested in seeing data but analysis depends on the physiotherapist	• Wears sensor 4x/year for a week • App only when the partner can manage • The physiotherapist views and analyzes the data and provides insights to PwPD during the consult	
**Persons not interested in monitoring their disease**					

Profile 1 represents people who are typically in an early phase of their PD, with good technical skills. They visit the physiotherapist a couple of times per year to proactively tackle small issues and stay physically active. For them, a monitoring system could act as a flagging dashboard. The objective sensor data could provide in-depth analyses of, e.g., gait parameters in daily life. In such parameters worsen, both the physiotherapist and PwPD could be notified and an appointment could be scheduled. In that way, the PwPD does not need to be in constant treatment so that overtreatment can be prevented while maintaining a reassuring view of the PwPD's status at home.

Profile 2 represents PwPDs who are typically in the mid-phase of their PD. They find it challenging to stay physically active and might experience near-fall incidents. For them, a monitoring system could add value as a motivational tool. For example, the PwPD and physiotherapist could set physical activity goals per week and use the sensor data to see if these goals were reached. Additionally, repeatedly collecting and discussing sensor data could increase awareness and understanding of important topics such as (near-)falls.

Profile 3 represents PwPDs who are typically in a mid-to-late phase of their PD. Their physiotherapy goals focus on managing (further) fall incidents and maintaining mobility to safely perform daily activities. For them, a monitoring system could serve as a supportive tool. These PwPDs start to experience cognitive impairments, which makes it difficult to remember, e.g., when, where, and why a fall occurred. A sensor could collect such objective information about falls and physical activity in the home situation. This information could be provided to the physiotherapist to optimize treatment.

Throughout the interviews, it became clear that monitoring systems are not adding value for all PwPDs. Some of the PwPDs said that they already know their PD well enough and do not need support in that. They were typically very early in their disease course and currently had limited physiotherapy-related issues. Other PwPDs had no interest in monitoring their disease in general. They did not wish to be constantly reminded of the disease through monitoring, as they often already struggled with accepting the disease in the first place. Finally, some PwPDs said that managing daily tasks was burdensome for them and they had no energy or time to deal with an additional system as well.

## Discussion

We designed and evaluated a remote monitoring system to support physiotherapy for PwPDs. Overall, both PwPDs and physiotherapists were positive about the usability and utility of the monitoring system for physiotherapy practice. Evaluating the usability and utility of any remote monitoring system is essential before implementation in real-life clinical practice is pursued. Specifically, for our system, physiotherapists see potential in objectively capturing physical activity and (near-)falls in daily life. The system motivated several PwPDs to move more because of the continuous and objective tracking of their physical activity. PwPDs and physiotherapists also enjoyed discussing the collected data. However, the system has clear improvement items before long-term implementation can be considered. For example, PwPDs and physiotherapists preferred automatic detection of a more diverse repertoire of activities, thereby minimizing the burden on the user.

Most PwPDs were capable of independently using the necklace and app at home without major issues. This is in line with another study suggesting that a majority of PwPDs can use technologies such as computers and smartphones in daily life (45). At the same time, we noticed that some participants got frustrated with the system. The system was too difficult for them, for example, because the system contained too many features or the PwPDs had few technical skills or slight cognitive impairments. We ensured that these PwPDs could also use and evaluate the system by offering a sensor-only option (i.e., merely passive recording) and we provided them with extensive remote technical support. Pursuing equal access to telehealth innovations requires constant attention as specific subgroups of PwPDs might be underrepresented in our research (46, 47). One possibility to increase equal access to innovations is to personalize the required user interactions with the tools. A modular system, for example, based around a smartphone can be designed to which different sensors can connect. Each person can then connect the sensors that best fit their needs and technical skills. Future studies are required to identify potential disparities in access to telemedicine and create specific solutions to mitigate these (48).

Several PwPDs emphasized the importance of the relationship with their physiotherapist. They looked forward to discussing the data with the physiotherapist, to seeing how they were doing, and to demonstrating the effort they had put into being more active. In turn, the physiotherapist encouraged the PwPDs to remain physically active and continue the use of the system. This finding is comparable to other literature that showed the importance of personal contact in adopting remote monitoring technology (49). Typically, when the amount of physical and social interaction with the physiotherapist or other group members decreased, the satisfaction with the therapy also decreased for the participants (31, 50). Other large-scale studies on the long-term adoption of sensor-based telemedicine have shown that compliance drops over time (24). This can be prevented or minimized when participants have a personal point of contact (25) and are motivated by relatives (9). The successful implementation of a teletreatment, therefore, strongly depends on a thorough understanding of the social context in which it is embedded.

Our study confirms that monitoring physical activity and falls is generally regarded as important (51, 52) but also confirms earlier impressions that a person-specific balance exists between the benefits and burdens of monitoring (53). All participants in our study used the same system which elicited highly divergent opinions. Some participants were not bothered by the necklace at all and were enthusiastic about the new insights they gained from the system. Others disliked wearing the necklace and felt the data were not accurate enough to be useful or did not want to be continuously reminded of their PD. Although the benefits of monitoring might never outweigh the burdens for some PwPDs, we strive to design inclusive monitoring systems useful for all PwPDs. Our user profiles describe this benefits–burdens balance for several groups of PwPDs but should be regarded as a starting point from which to explore even more personalized monitoring needs and wishes. For example, the profiles could be combined with other known benefits and burdens of monitoring, (53, 54) physiotherapy treatment mechanisms (4), and personality traits such as coping (55, 56) and information-seeking styles (57). Drafting user profiles of physiotherapists could help to create systems that also accommodate their needs and preferences.

The strength of this study is the unique insight gained from daily practice about how a sensor-based monitoring system can support physiotherapy. We had an extensive study period duration of 6 weeks, allowing for substantive wear and use periods leading to grounded conclusions by the participants. By deploying an iterative design process, we could intermediately incorporate the feedback from PwPDs and physiotherapists to improve the system.

However, this study was not without limitations. First, the SUS was lower in pilot 2 despite seeming improvements in the system and increased compliance. An explanation could be that the added features of the system also made the system more complex. As these features were not readily used, this could decrease the usability of the system. Another explanation could be that we recruited more affected persons with PD in pilot 2 who experienced more difficulties with operating the system. To be able to elaborately test the fall section of the system, we specifically recruited more persons with PD who experienced (near) falls in pilot 2 (Table 2). Most likely as a consequence of our recruitment strategy, the pilot 2 participants have worse scores on all clinical outcomes compared to pilot 1 participants, except for the Hoehn and Yahr stage, which is difficult to accurately classify. Furthermore, the SUS could be lower because we encountered some technical problems in pilot 2 such as data not showing in the app. Based on the user feedback in pilot 1, we increased the available technical support for pilot 2. This support was appreciated and ensured that people were retained in the study. In total, only three participants dropped out during both pilots because the app was too difficult for them or because they were frustrated by the lack of correctable data.

Second, the user profiles were only indirectly assessed within the interviews since the interviews were specifically aimed at evaluating the system. However, we grounded the user profiles as much as possible in the available data through a rigorous analysis, including discussions with the research team. Future research should focus on further developing these profiles, for example, by refining their content and applicability through co-creation sessions with PwPDs and physiotherapists. Furthermore, we drafted these profiles to understand how monitoring tools could add value for specific subgroups of PwPDs by generalizing people's similarities. We are aware that each PwPD is unique and has their own contexts and wishes, so PwPDs may or may not find resemblances in our profiles.

Third, our sampling method poses limitations on the generalizability of our findings regarding both physiotherapists and PwPDs. The physiotherapists taking part in our study were all part of ParkinsonNet in the Netherlands and, as such, were thoroughly trained in treating PwPDs (32). Being part of the Dutch ParkinsonNet also means that the participating physiotherapist will attract a much higher caseload, which will presumably also help as an encouragement to start using a new technological system for that specific population, unlike more generically trained therapists who only sporadically encounter PwPDs in their practice. In other countries, the role of the physiotherapist in the treatment of PwPDs might be different, instigating different usability and utility evaluations. However, the high quality of specialized Parkinson-specific physiotherapy does make the Netherlands a suitable test climate for the development and evaluation of such tools. Regarding the PwPDs, a selection bias might have occurred because they were selected from the database of the physiotherapist. Physiotherapists might have invited participants who, for example, have an above-average affinity with technology. We partly mitigated this problem in pilot 2 by allowing participants to only use the sensor if using the app was too complicated. Still, our sample most likely contains PwPDs who are interested in monitoring technology or healthcare innovations in general. Testing the system in these PwPDs leads to relevant conclusions as they are also most likely to adopt monitoring systems. However, this also means that our findings might not generalize to a broader PD population for whom monitoring tools will also become accessible in the future.

Our study has shown that physiotherapists and PwPDs are interested in sensor-based data, but our system requires further development and testing before it is ready for actual implementation in clinical practice. The development of the system should focus on improving its technical maturity as well as expanding its functionalities, which should be driven by specific use cases for remote monitoring and individual characteristics of the users. We organized our findings related to this in different user profiles, which can guide future development. Specifically for PwPDs, future tools should become more adjustable for each person. For example, PwPDs should be able to choose whether they see the same detailed data as the physiotherapist or only receive high-level summaries. Also, automatically detecting more diverse physical activities is important to reduce the burden of the tool. Yet, adding more subjective measures such as feelings and motivations should be possible as they give context to the objective data (Table 2). Specifically, for physiotherapists, the treatment of falls could be supported by providing them with more sensor-based indicators of fall risk, e.g., a more in-depth analysis of the free-living gait pattern and transfers. Finally, rigorous testing is needed to establish the added value of this sensor-based information for clinical practice (58). After developing such matured systems, future research should examine the long-term effect of monitoring systems on therapy decision-making, their affect on quality of life, and their cost-effectiveness, all within well-defined target populations.

## Data availability statement

The datasets presented in this article are not readily available because participants did not agree to the public sharing of their data. Requests to access the datasets should be directed to LE, luc.evers@radboudumc.nl.

## Ethics statement

The studies involving humans were approved by Commissie Mensgebonden Onderzoek regio Arnhem-Nijmegen. The studies were conducted in accordance with the local legislation and institutional requirements. The participants provided their written informed consent to participate in this study.

## Author contributions

RB, LE, ND, AS, BB, and MM were responsible for the conception of the research idea and design of the study. ND, BB, GV, and MM obtained the funding for the study. All authors were involved in the analysis and interpretation of the data. RB and LE drafted the initial manuscript, which was thoroughly reviewed by the other authors. All authors read and approved the final version of the manuscript.

## Supplementary material

Supplementary Figure S1 The Vital@Home physiotherapist app in pilot 1 **(Figures S1 and S2)** and pilot 2 **(S3-S7)**, including an overview of physical activities **(S1)**, an overview of (near-)falls **(S2)**, the renewed physical activity **(S3 and S4)** and fall **(S5)** overview, the personalized exercise program **(S6)**, and the gait analysis section **(S7)**.
