# Supplementary figures and images for: Usability and utility of a remote monitoring system to support physiotherapy for people with Parkinson's disease
Source: Front Neurol. 2023 Oct 12;14:1251395. doi: 10.3389/fneur.2023.1251395 (PMC10601712; doi:10.3389/fneur.2023.1251395)

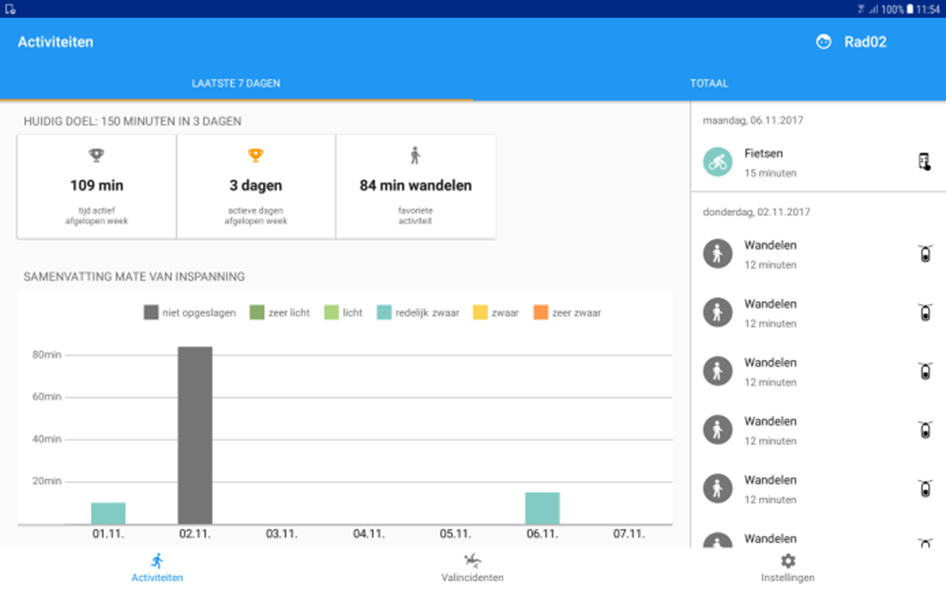

Supplement: Supplementary Figure S1 — The Vital@Home physiotherapist app in pilot 1 (Figures S1 and S2) and pilot 2 (S3-S7), including an overview of physical activities (S1), an overview of (near-)falls (S2), the renewed physical activity (S3 and S4) and fall (S5) overview, the personalized exercise program (S6), and the gait analysis section (S7). [file Image_1.PNG]

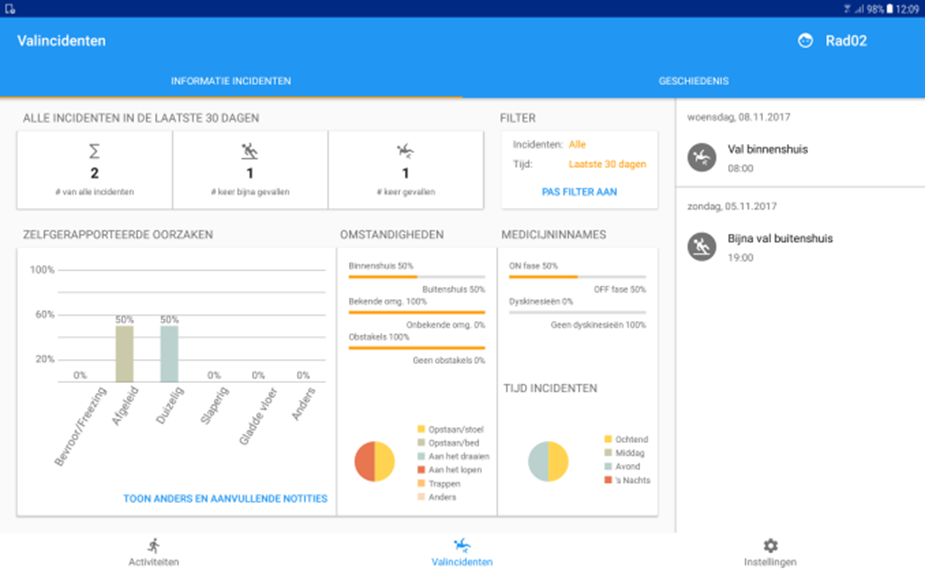

Supplement: Supplementary file 2 [file Image_2.PNG]

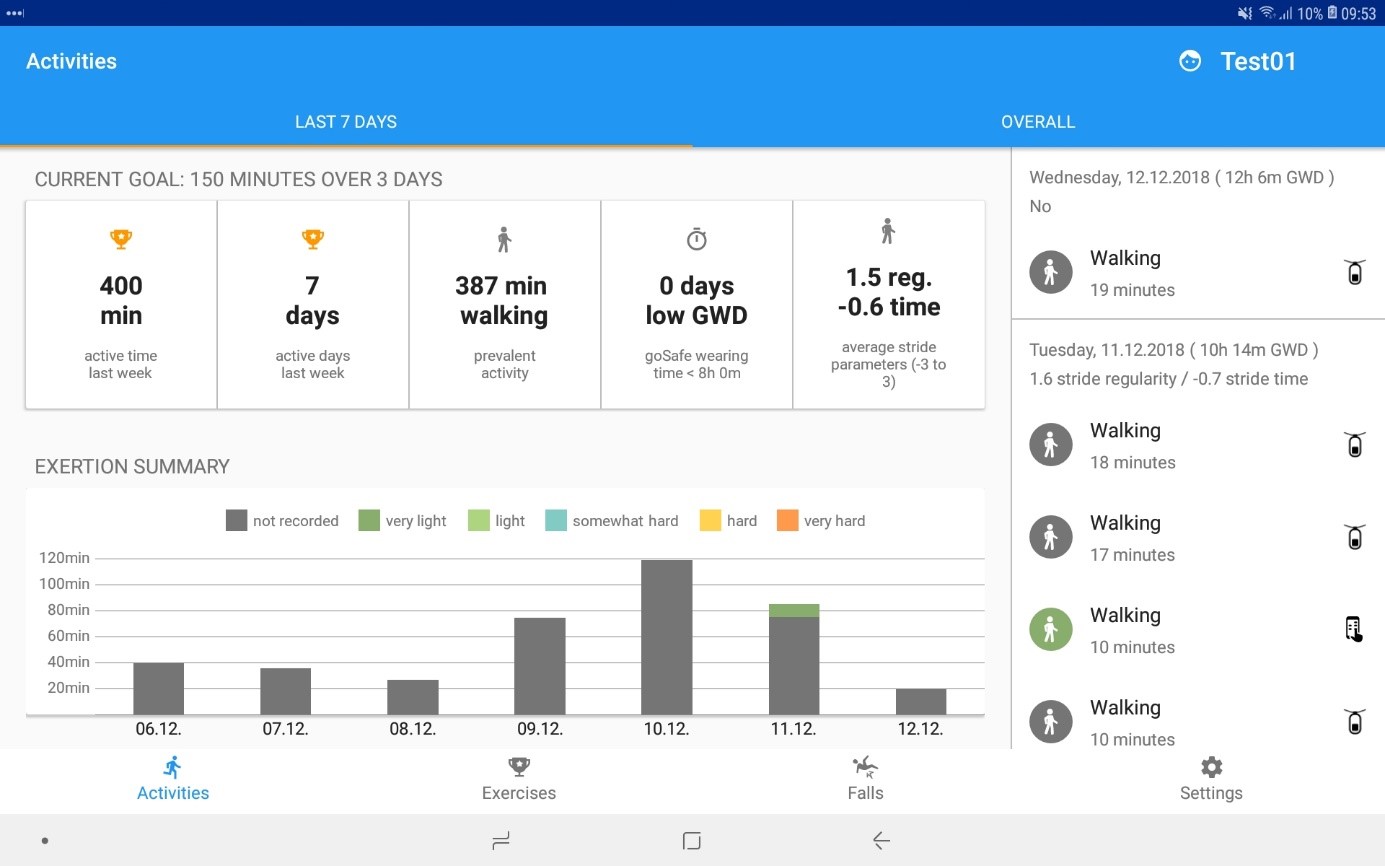

Supplement: Supplementary file 3 [file Image_3.JPEG]

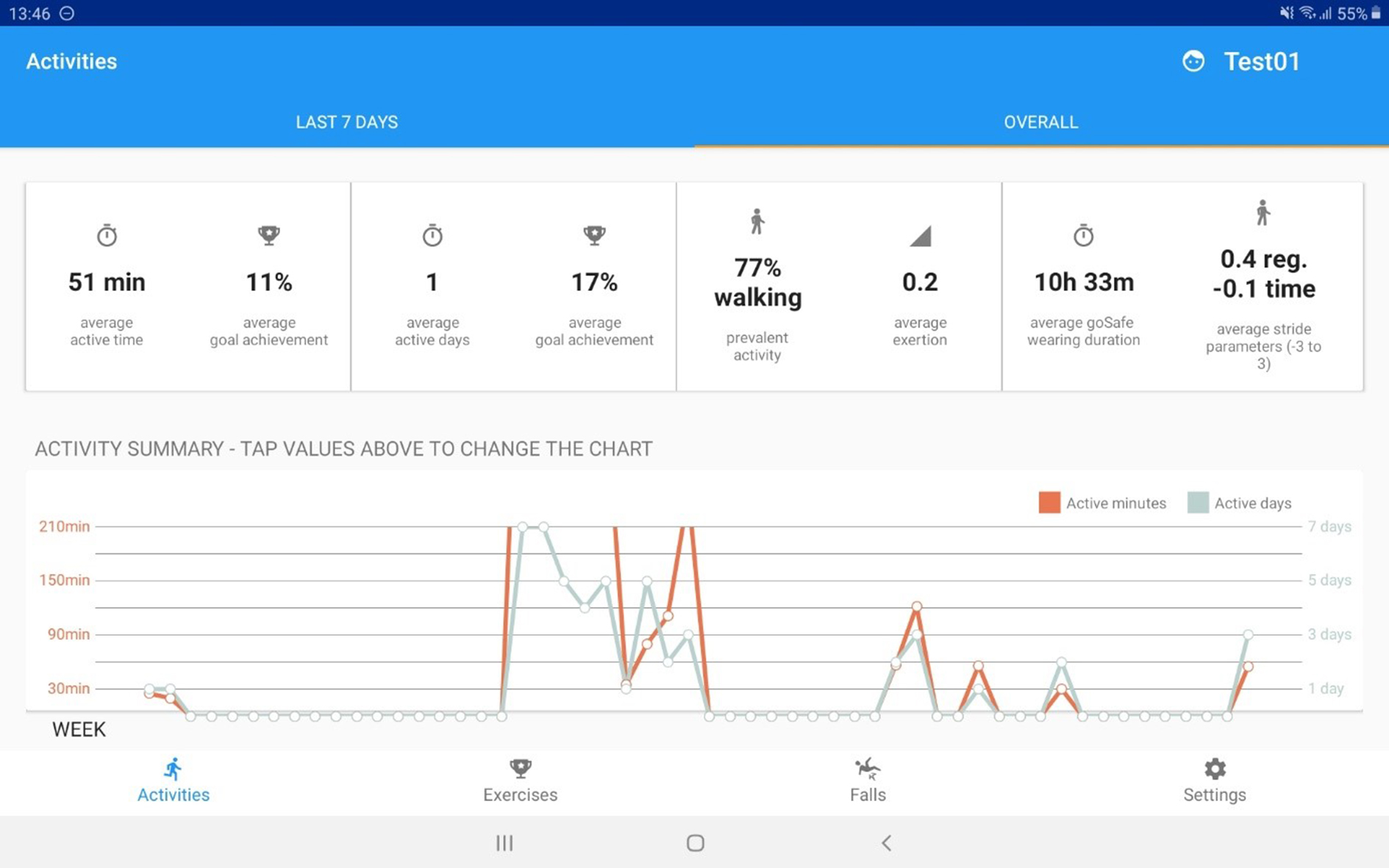

Supplement: Supplementary file 4 [file Image_4.JPEG]

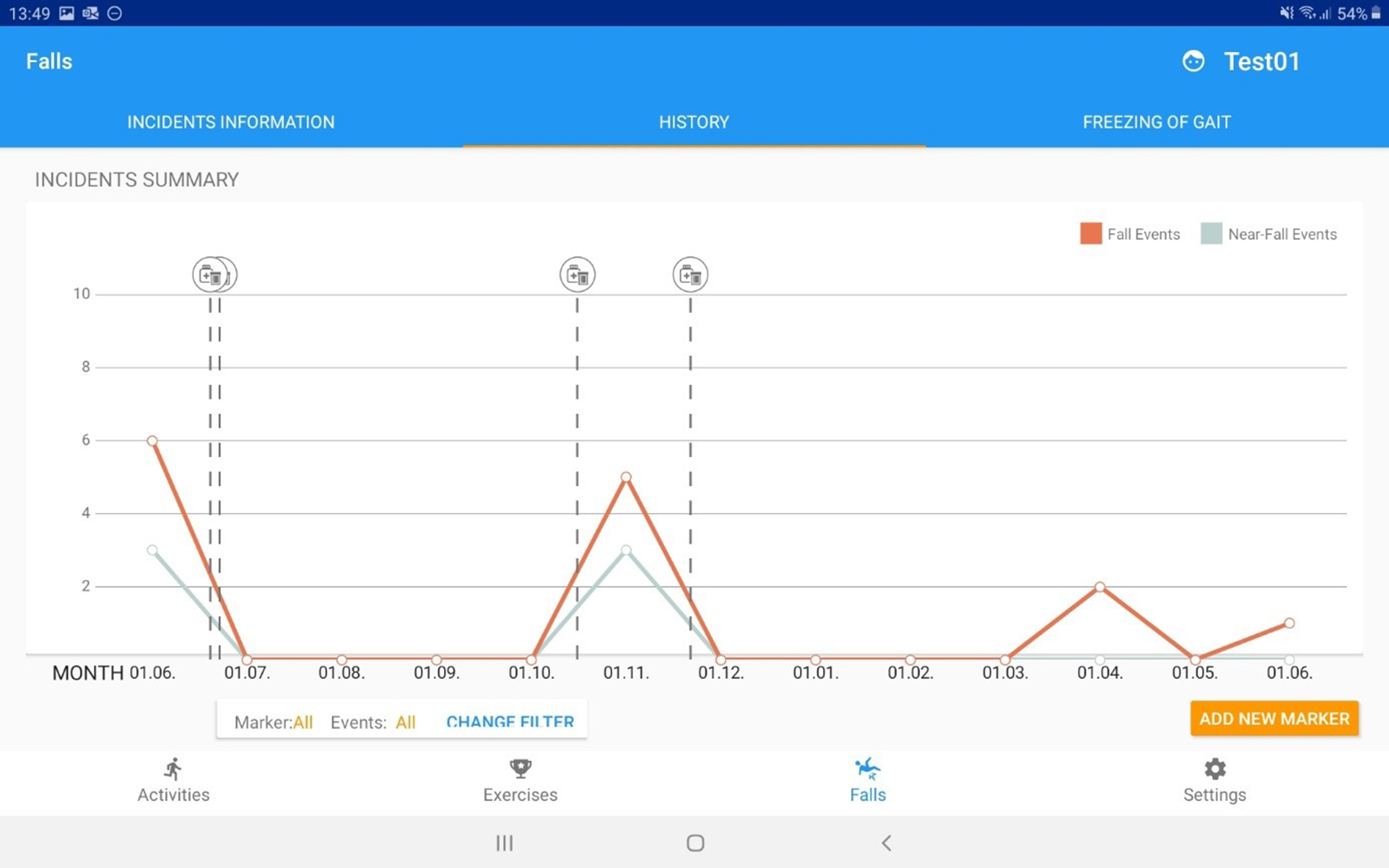

Supplement: Supplementary file 5 [file Image_5.JPEG]

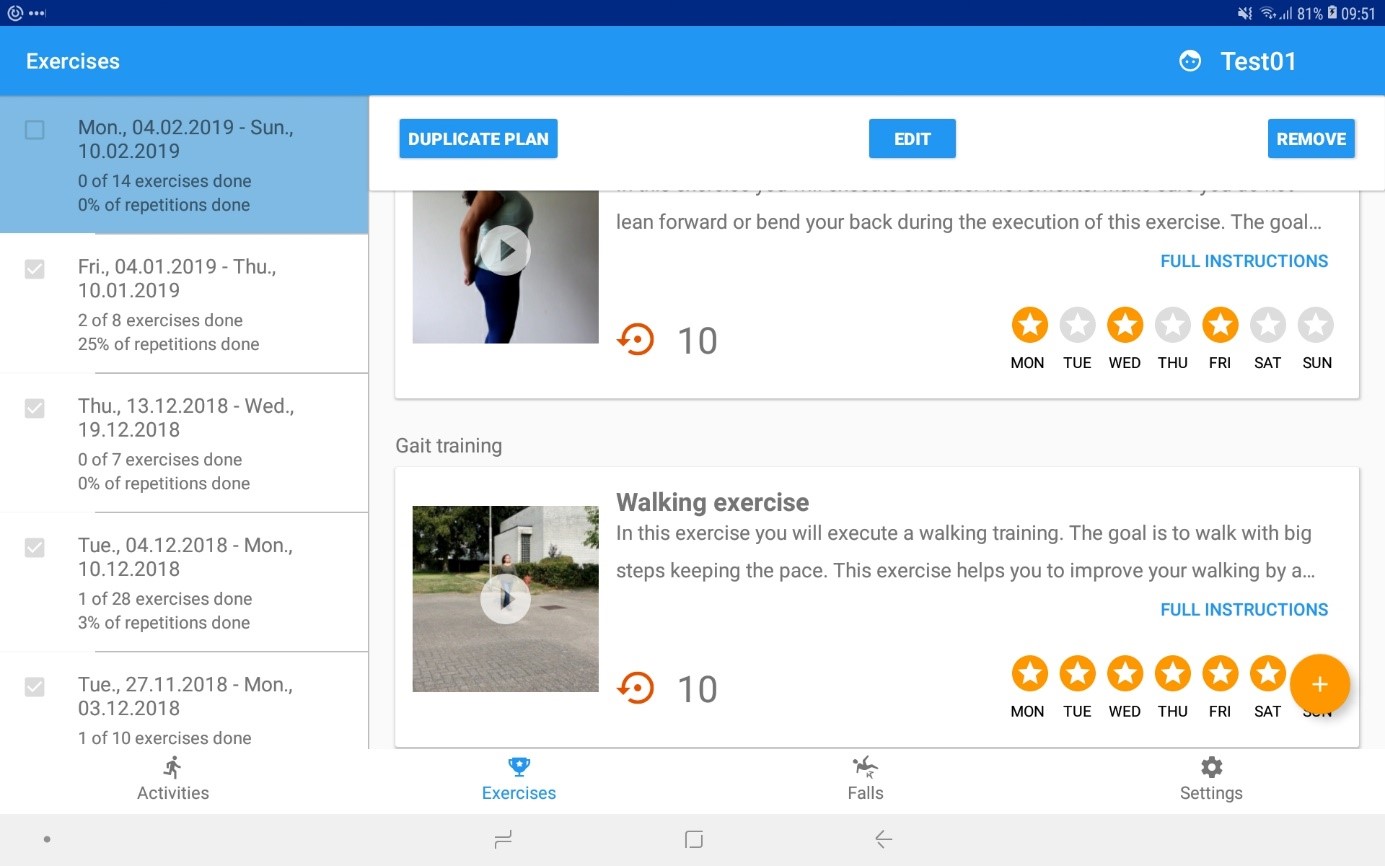

Supplement: Supplementary file 6 [file Image_6.JPEG]

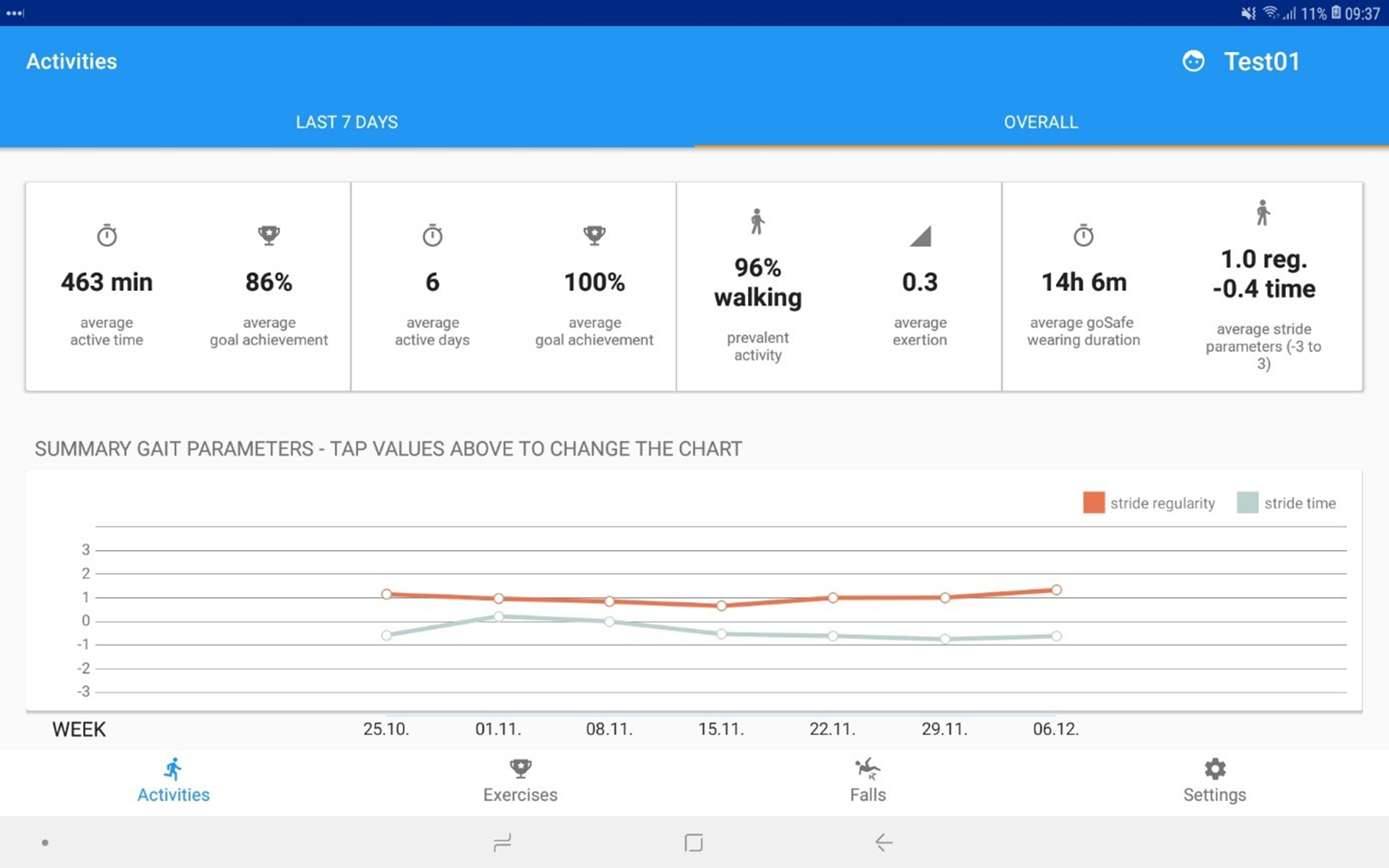

Supplement: Supplementary file 7 [file Image_7.JPEG]
